# Genetic diversity and phylogeographic dynamics of avihepadnavirus: a comprehensive full-length genomic view

**DOI:** 10.3389/fvets.2024.1385033

**Published:** 2024-05-02

**Authors:** Muhammad Sikandar, Pir Tariq Shah, Li Xing

**Affiliations:** ^1^Institute of Biomedical Sciences, Shanxi University, Taiyuan, China; ^2^Faculty of Medicine, School of Biomedical Engineering, Dalian University of Technology, Dalian, China; ^3^Shandong Laboratory of Yantai Drug Discovery, Bohai Rim Advanced Research Institute for Drug Discovery, Yantai, China; ^4^Shanxi Provincial Key Laboratory of Medical Molecular Cell Biology, Shanxi University, Taiyuan, China

**Keywords:** avihepadnavirus, phylogenetic, phylogeographic, recombination, genetic evolution

## Abstract

Avihepadnavirus is a genus of the Hepadnaviridae family. It primarily infects birds, including species of duck, geese, cranes, storks, and herons etc. To understand the genetic relatedness and evolutionary diversity among avihepadnavirus strains, a comprehensive analysis of the available 136 full-length viral genomes (*n* = 136) was conducted. The genomes were classified into two major genotypes, i.e., GI and GII. GI viruses were further classified into 8 sub-genotypes including DHBV-I (duck hepatitis B virus-I), DHBV-II (Snow goose Hepatitis B, SGHBV), DHBV-III, RGHBV (rossgoose hepatitis B virus), CHBV (crane hepatitis B virus), THBV (Tinamou hepatitis B virus), STHBV (stork hepatitis B virus), and HHBV (Heron hepatitis B virus). DHBV-I contains two sub-clades DHBV-Ia and DHBV-Ib. Parrot hepatitis B virus (PHBV) stains fall into GII which appeared as a separate phylogenetic branch/clade. All the subtypes of viruses in GI and GII seem to be genetically connected with viruses of DHBV-I by multiple mutational steps in phylogeographic analysis. Furthermore, 16 potential recombination events among different sub-genotypes in GI and one in GII were identified, but none of which is inter-genotypic between GI and GII. Overall, the results provide a whole picture of the genetic relatedness of avihepadnavirus strains, which may assist in the surveillance of virus spreading.

## Introduction

In the family of *Hepadnaviridae*, viruses are classified into five genera based on the host species, i.e., *Avihepadnavirus*, *Orthohepadnavirus*, *Parahepadnavirus*, *Metahepadnavirus*, and *Herpetohepadnavirus*. *Avihepadnavirus*, such as the Avian hepatitis B-type virus, specifically infects birds. *Orthohepadnavirus* includes human hepatitis B virus (HBV) and other viruses infecting mammals. *Parahepadnavirus, Metahepadnavirus,* and *Herpetohepadnavirus* usually infect reptiles, amphibians, and aquatic species, respectively (1). To date, the genus *Avihepadnavirus* comprises of viruses infecting various species of avian, including duck (duck hepatitis B virus, DHBV), geese (Snow goose hepatitis B, SGHBV and Ross goose hepatitis B virus, RGHBV) (2), cranes (Crane hepatitis B, CHBV) (3), herons (Heron hepatitis B DHBV) (4), storks (Stork Hepatitis B virus, STHBV) (5), and parrots (Parrot hepatitis B virus, PHBV) (6). According to the International Committee on Taxonomy of Viruses (ICTV) classification, the *Avihepadnavirus* genus is composed of three main species based on nine avihepadnavirus polymerase gene sequences: *Duck hepatitis B virus*, *Crane hepatitis B virus*, and *Perrot hepatitis B virus* (accessed on April 17, 2024).^1^ Notably, *Duck hepatitis B virus* also encompasses RGHBV, CHBV (3), Sheldgoose Hepatitis B virus, and Snow Goose Hepatitis B virus. *Crane hepatitis B virus* (4) also includes another species called STHBV. In that avihepadnavirus classification, *Perrot hepatitis B virus* is placed in a separate third category. Additionally, there is an unidentified species known as the *Elegant-Crested Tinamou Hepatitis B virus*, which has not been assigned to any of the three main species of *Avihepadnavirus* (1).

Avihepadnavirus has a circular, partly double-stranded DNA (dsDNA) genome of about 3.0 kb in length. Viral infection can cause liver injury, cirrhosis, or the development of hepatocellular carcinoma (HCC) (7). As the replication of hepadnaviruses is carried out by reverse transcription of the RNA pre-genome, the liver of infected animals always contains a variety of populations of DNA and DNA–RNA duplex (8). The circular viral genomes of avihepadnavirus are characterized by several overlapping open reading frames (ORFs) that encode polymerase (P), core (C), and surface (S) proteins, which are responsible for virus replication and viral particle assembly (9). P protein functions as reverse transcriptase, DNA-dependent DNA polymerase, and RNase H enzyme. During the replication of viral genome, the P protein, also known as the viral polymerase, is essential for initiating the synthesis of complementary strand DNA (10) and ensures the proper repair of DNA gaps to enable virus to replicate efficiently (11). The C protein of hepadnaviruses is responsible for the encapsulation of viral genome to form nucleocapsid. This protein safeguards viral genomic DNA throughout different stages of virus life cycle (12). The preS/S protein is necessary for the assembly of virion and virus-host interaction (13). This protein is situated on the virus envelope and plays a critical role in the entry of virus into host cells and also contributes to immune recognition (14). Among avihepadnavirus-encoded proteins, the C protein is relatively more conserved (15), but the S protein exhibits higher antigenicity. The variability observed in S protein contributes to their antigenic diversity (8).

Among avihepadnavirus, DHBV received more research attention. DHBV was initially identified from Pekin ducks in China (16) and later identified in different geographical areas including the United States (16), Australia (17, 18), Germany (19), and South Africa (20). In terms of genetic organization, virus replication, and biological characteristics, DHBV is like HBV (21). However, breeding ducks may experience a persistent infection of DHBV, which can cause significant damage to duck hatching and growth (22). Previous research has shown that a single amino acid change in P protein can affect subsequent liver damage (23). These findings indicate that DHBV constitutes a significant threat to poultry health. Thus, understanding of genetic relatedness and evolutionary diversity of avihepadnavirus strains identified from different host animal species in different country can provide valuable information for the surveillance of virus spreading.

## Genetic relatedness of avihepadnavirus strains

**Figure 1:**
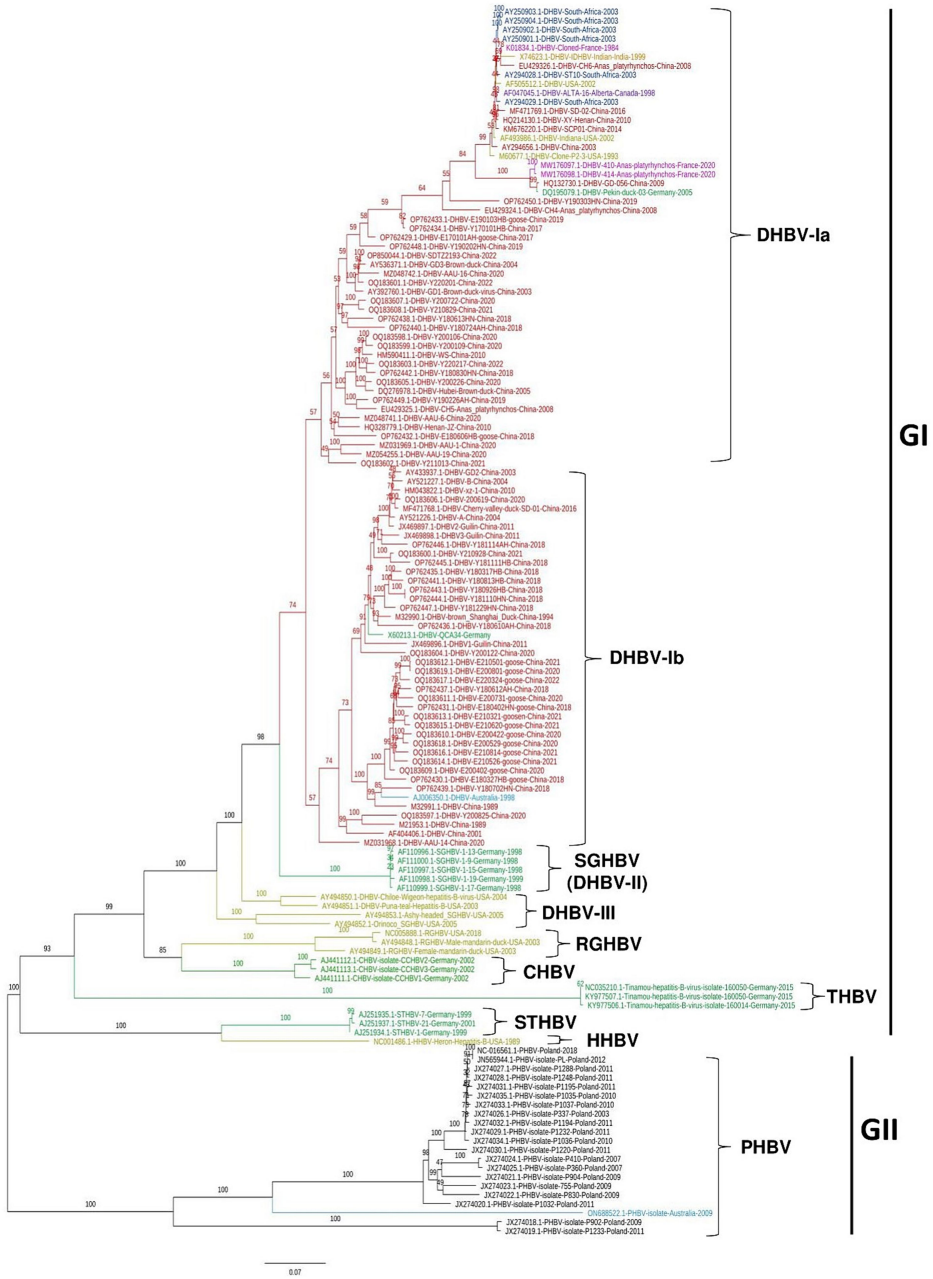
Phylogenetic tree of avihepadnavirus full-length genomes. A Maximum Likelihood (ML) phylogenetic tree of 136 full-length genome sequences of avihepadnavirus was inferred using the IQ-TREE v1.6.12 with best-fitting model TPM2u + F + I + G4 and 1,000 bootstraps. Each color represents a different country. The tree was visualized and modified using FigTree v1.4.

To determine the genetic relatedness of avihepadnavirus identified from different animal species including Duck, Snow goose, Ross’s goose, Crane, Heron, Stork, and Parrot et al., a total of 136 complete genome sequences of avihepadnavirus were retrieved from NCBI GenBank, which were identified in 9 countries including China (*n* = 76), Germany (*n* = 16), United States (*n* = 11), France (*n* = 3), South Africa (*n* = 6), Australia (*n* = 2), India (*n* = 1), Canada (*n* = 1) and Poland (*n* = 20). The sequences were aligned and then trimmed using BioEdit v7.2.5 (24). In our analysis, the sequence was trimmed mainly based on the full-length sequence of virus strain DHBVQCA34 (GenBank ID: X60213.1) as the reference sequence in the dataset. The Maximum likelihood (ML) phylogenetic tree was constructed using the IQ-TREE v1.6.12 with the best-fitting model TPM2u + F + I + G4 (25). As shown in Figure 1 and Supplementary Table S1, the viruses were classified into two major clades, GI and GII. GI was further divided into 8 sub-clades, i.e., DHBV-I, DHBV-II (SGHBV), DHBV-III, RGHBV, CHBV, THBV, STHBV, and HHBV, whereas GII appeared as an independent clade, PHBV. Each sub-clade represents a genetically distinct group. DHBV-I can be further divided into DHBV-Ia and -Ib. The ML phylogenetic tree was also constructed based on the S protein gene (Supplementary Figure S1). This tree is roughly consistent with the results of full-length genome-based tree, but the obvious differences were found for viruses among DHBV-Ia, DHBV-Ib, and DHVB-II, where DHVB-II is relocated between DHBV-Ib and DHBV-Ia (Supplementary Figure S1), indicating that the S genes of those viruses are genetically closer to each other.

Viral strains identified in China were sorted in DHBV-Ia and -Ib in GI. Among 51 strains of DHBV-Ia, 36 strains were identified in China from 1989 to 2022. Remaining virus strains in DHBV-Ia were identified from different countries including the United States (*n* = 3), South Africa (*n* = 6), France (*n* = 3), India (*n* = 1), Germany (*n* = 1) and Canada (*n* = 1). In DHBV-Ib, 40 out of 42 strains were identified from China and one strain was identified in Australia in 1998 (GenBank ID: AJ006350.1) (18) and another one was reported in Germany in 1991 (GenBank ID: X602213.1-DHBV). DHBV-Ia and -Ib represent the current sub-genotypes of DHBV prevalent in China. Five SGHBVs identified in Germany (2) form an independent sub-clade DHBV-II. Four viruses identified in United States from different animals including Chiloe Wigeon (GenBank ID: AY494850.1), Puna-teal (GenBank ID: AY494851.1), Ashy-headed sheldgoose (GenBank ID: AY494853.1), and Orinoco sheldgoose (GenBank ID: AY494852.1) were genetically closer to each other and thus classified as DHBV-III (Figure 1). RGHBV (26), CHBV, THBV, STHBV (5), and HHBV (4) are genetically far from each other and form different sub-clades in phylogenetic tree, which may suggest the unique evolutionary trajectory and genetic differentiation. The viruses of GII clade are PHBV, which were identified from parrots. So far, CHBV, THBV, and STHBV have been identified only in Germany. HHBV and RGHBV were reported only in the United States (Figure 1).

Since the avihepadnavirus shows greater genetic diversity among different clades and sub-clades, the genetic similarity of representative sequences from each sub-clade was determined using SimPlot v3.5.1 (27) with a strain of DHBV-Ia identified in China in 1989 (GenBank ID: OP762450.1) as a query sequence (Supplementary Figure S2). The genomic region encoding the N-terminal part of S protein (nt 600–1,300) shows the lowest similarity (<80%), whereas the genomic region encoding the C-terminal part of S protein (nt 1,300–1800) is highly conserved (>85%).

## Phylogeographic network of avihepadnavirus strains

**Figure 2:**
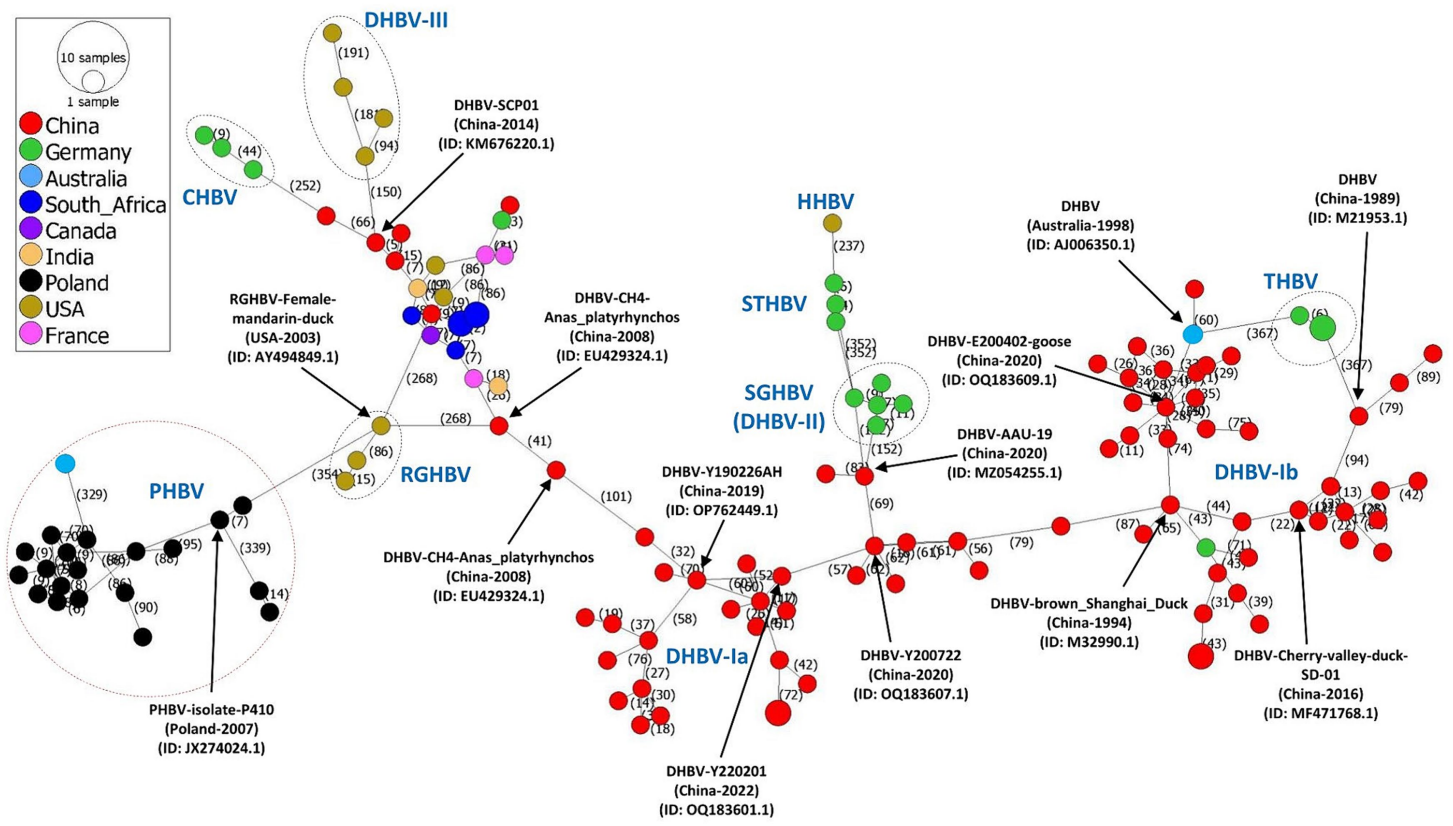
Phylogeographic network based on full-length genome sequences of avihepadnavirus. The phylogeographic analysis of 136 full-length genome sequences of avihepadnavirus was performed using the MSN implemented by PopArt v1.7. Each node represents one virus strain. The distance between the nodes represents the number of mutational steps. Each color represents a different country.

In the finding of mutational steps and spread of avihepadnavirus, the phylogeographic pattern of avihepadnavirus was mapped by inferring the minimum spanning network (MSN) using the evolutionary software PopArt v1.7 (28) (Figure 2). The results reveal multiple mutational branches. The virus strains in DHBV-Ia and -Ib connected genetically with most of other sub-genotypes. For example, SGHBVs identified in Germany seem to be derived from virus strain AAU-19 (DHBV-Ia, GenBank ID:MZ054255.1) by 152 mutational steps. Additional mutation steps on the basis of SGHBV leads to the emergence of STHBV and HHBV. DHBV-III viruses and CHBVs can originate from DHBV-Ia virus SCP01 (GenBank ID: ID: KM676220.1) by at least 150 and 318 mutation steps, respectively. One RGHBV strain (GenBank ID: AY494849.1) identified in the United States in 2003 is responsible for the emergence of multiple viral sub-genotypes identified worldwide, including DHBV-Ia strains and PHBV strains by 268 and 354 mutational substitutions, respectively (Figure 2). Overall, the phylogeographic analysis results also indicate the great diversity among avihepadnavirus and support the phylogenetic analysis in that genetic features of viruses are closely related to geographical areas.

## Genomic recombination of avihepadnavirus

To evaluate the genetic exchanges between avihepadnavirus strains, 136 complete genome sequences were analyzed using seven algorithms (RDP, MaxChi, Chimera, Bootscan, GENECONV, SiScan, and 3seq) embedded in the RDP4 software package with various statistical measures including *p*-values and confidence intervals to verify the potential recombination events (29). A recombination event identified by at least four of seven algorithms in RDP4 was accepted as potentially real. A total of 17 potential recombination events were confirmed by at least four of seven algorithms for each (Supplementary Table S2). One recombinant PHBV virus (GenBank ID: ON688522.1) was generated through recombination between PHDV strains in GII. One representative minor parent in this recombination was PHBV isolate P902 (GenBank ID: JX274018.1), while the representative major parent was PHBV isolate P1032 (GenBank ID: JX274020.1)(Event 10). All remaining recombination events occurred within or between sub-genotypes DHBV-Ia, DHBV-Ib, or RGHBV in GI. We did not detect recombination between GI and GII. Noticeably, the virus strains identified in China dominate both the dataset of this study and recombination events. The recombination occurred throughout the whole viral genome (Supplementary Figure S3). For example, the breakpoints of events 3, 4, 5, 7, 8, 12, 15, and 17 were found in S- and P-ORF regions, while that of events 1, 2, 6, 9, 10, 11, and 13 were between C- and P-ORF regions; Event 14 was found in the C-ORF region.

## Amino acid variability pattern of avihepadnavirus proteins

To see the potential antigenic drift, we analyzed the variability of amino acids in three different proteins of avihepadnavirus, i.e., P, C, and S proteins (Supplementary Figure S4). The complete nucleotide sequences of ORFs encoding these proteins were retrieved from the NCBI database, aligned, and translated into amino acid sequences using MEGA11 software (30, 31). Amino acid variability was achieved using the Wu-Kubat variability coefficient method implemented in the Protein Variability Server (PVS) (32). The variability coefficient was calculated using the following formula: variability = n*k/N, where n is the number of sequences, k is the number of different amino acid at a given position, and N represents the number of most recognized amino acids at a specific position. The reference sequences for P, C, and S proteins are 450, 200, and 100 amino acids, respectively. Among three proteins, P contains multiple higher mutation regions (aa 10–25, 285–295, 370–380, 440–450). C and S proteins are relatively conserved. These variations in amino acids further indicate the diversity and adaption of avihepadnavirus genome.

## Discussion

DHBV along with a number of DHBV-like viruses including SGHBV, RGHBV, CHBV, THBV, STHBV, HHBV, and PHBV identified from different avian species were placed in the genus *Avihepadnavirus* in the family *Hepadnaviridae* (1, 33). However, the classification of avihepadnavirus at the genotype and sub-genotype levels in different studies shows significant discrepancies. For example, avihepadnavirus was sorted into two main groups, i.e., Chinese identified strains and Western identified strains in a study of 30 partial genome sequences (26). Ji et al. classified the avihepadnavirus DHBV strains into three main clades named as DHBV-Ia (Chinese strain), DHBV-Ib (Chinese strain), and Western DHBV-II strain (34). The differences in avihepadnavirus classification proposed by previous studies are related to different methods and reference sequences (26, 35). Therefore, it is necessary to analyze all the available complete genomic sequences of avihepadnavirus strains together to obtain the most robust results. Phylogenetic and phylogeographic analysis of all available complete genomic sequences of avihepadnavirus revealed two genotypes GI and GII. GI contains 9 sub-genotypes which were defined as DHBV-Ia, DHBV-Ib, DHBV-II (SGHBV), DHBV-III, RGHBV, CHBV, THBV, STHBV and HHBV. So far, the viruses of DHBV-I dominate the genus *avihepadnavirus.* Particularly, DHBV-Ia viruses were widely distributed in different continents including Asia, Africa, Europe, and North America.

Genetic recombination is crucial in the evolution of viruses and plays a significant role in generating the genetic diversity of viruses for fitness. A few of recombination events were reported for avihepadnavirus strains (6). The additional recombination events identified in our analysis indicate that recombination is one major factor driving the evolution of avihepadnavirus.

It is important to acknowledge that some studies have reported partial sequences of avihepadnavirus from various countries (36). However, we focused on conducting a comprehensive analysis of full genome sequences of avihepadnavirus from different regions around the world to gain a holistic understanding of the genetic characteristics and evolutionary relationships of these viruses. Those partial sequences contribute to the collective knowledge in the field and can be valuable resources for future research while it does not fit in our analysis.

Overall, this study provides insight into the evolutionary relationship between the avihepadnavirus and the diverse characteristics of each species, which are informative for the development of vaccines, surveillance, and improvement of diagnostic instruments in curbing the spread of viruses.

## Data availability statement

The nucleotide sequence data used in this study are available in NCBI GenBank.

## Ethics statement

For this retrospective type of study, formal consent is not required. Statement on the welfare of animals is not applicable as sample collection from animals has been done before.

## Author contributions

MS: Formal analysis, Writing – original draft. PT: Data curation, Formal analysis, Investigation, Visualization, Writing – review & editing. LX: Conceptualization, Funding acquisition, Project administration, Supervision, Writing – original draft, Writing – review & editing.

## Footnotes

^1^ https://ictv.global/report/chapter/hepadnaviridae/hepadnaviridae
