# Supplementary material for: Genetic diversity and phylogeographic dynamics of avihepadnavirus: a comprehensive full-length genomic view
Source: Front Vet Sci. 2024 May 2;11:1385033. doi: 10.3389/fvets.2024.1385033 (PMC11096447; doi:10.3389/fvets.2024.1385033)

Supplementary Figure S1. Phylogenetic tree of full-length S protein ORF of avihepadnavirus. A Maximum Likelihood (ML) phylogenetic tree of 136 S protein ORF sequences of avihepadnavirus was inferred using the IQ-TREE v1.6.12 with best-fitting model GTR+F+I+G4 and 1000 bootstraps. Each color represents a different country. The tree was visualized and modified using FigTree v1.4.

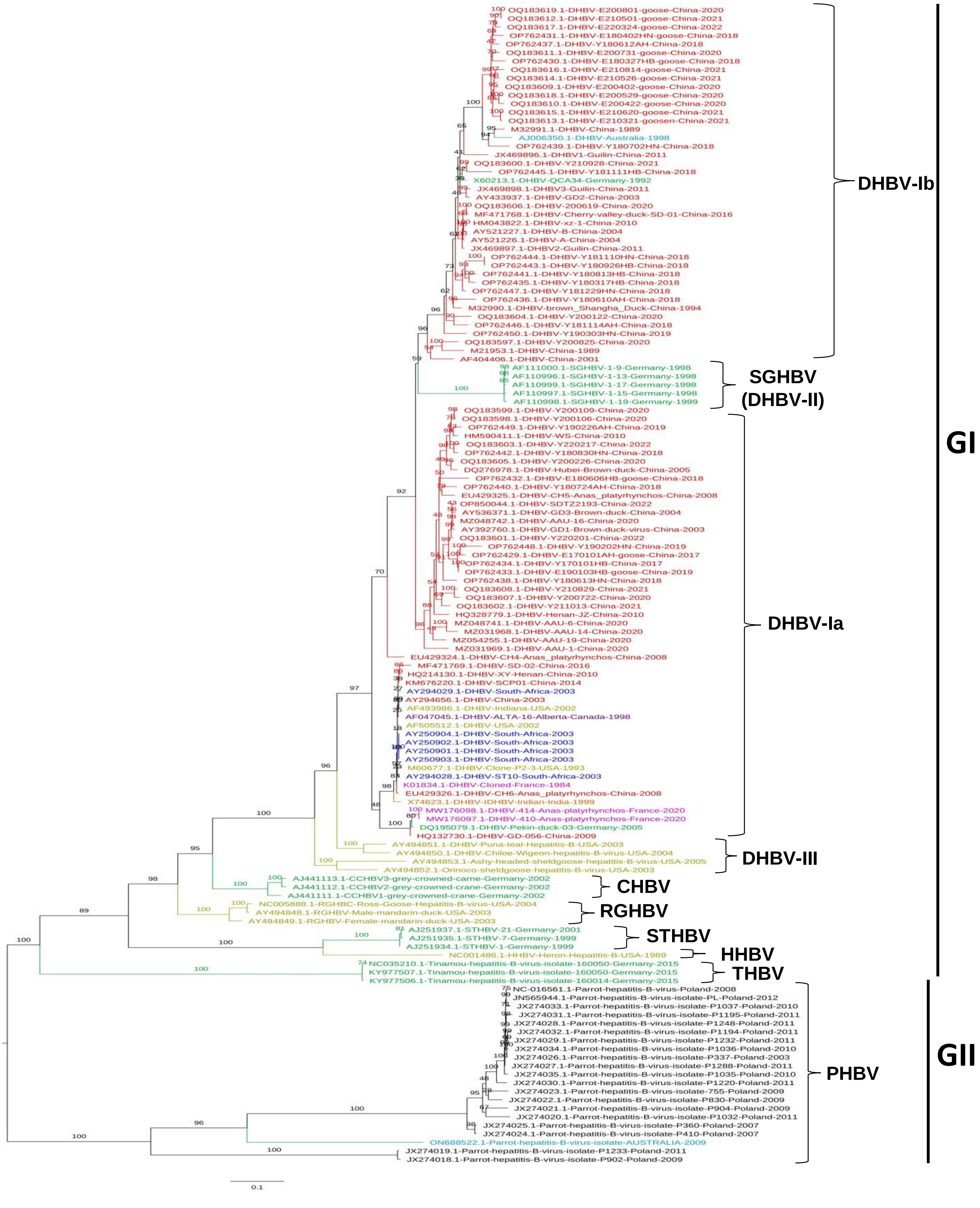

Supplementary Figure S2. Genomic similarity analysis of representative viruses selected from each sub-genotypes of avihepadnavirus. (A) Linear genomic structure of the avihepadnavirus including the ORFs encoding C, P, and S proteins. (B) Ten representative avihepadnavirus strains from each sub-genotype in the phylogenetic tree of Figure 1 were compared using genomic similarity plot carried out with SimPlot ver.3.5.1. The X-axes shows the nucleotide similarity percentage, and the Y-axes shows the nucleotide position. The similarity is presented in percentage (%). The genome of virus DHBV Y190303HN (GenBank ID: OP762450.1) was used as a Query.

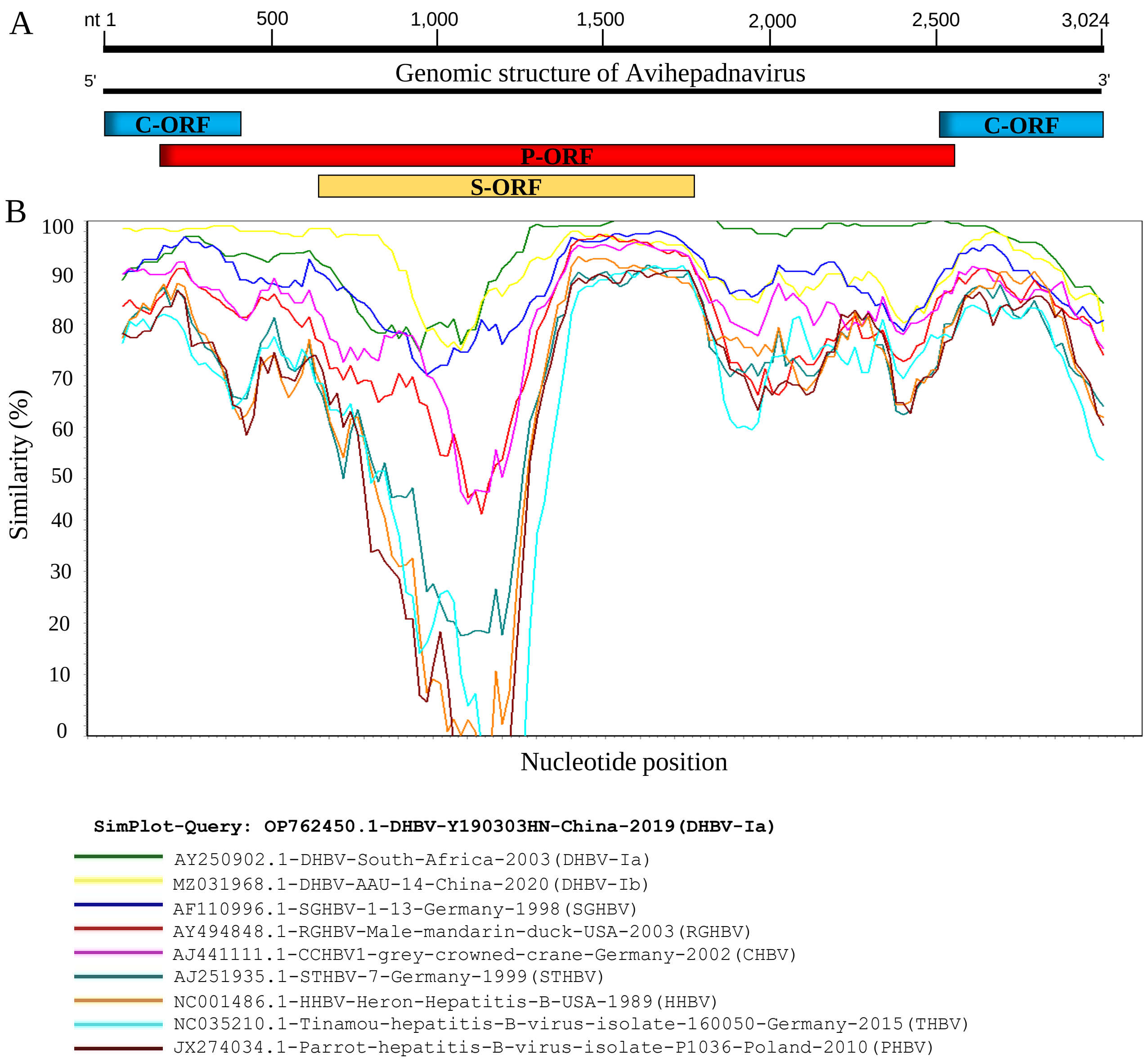

Supplementary Figure S3. Genetic recombination analysis of 136 full-length avihepadnavirus genomes. (Upper part) Diagram showing the full-length genome of avihepadnavirus and the corresponding regions encoding C, P, and S proteins. (Lower part) Schematic representation of the potential recombination events listed in Supplementary Table S2. Recombination event serial numbers and the description of potential recombinants (GenBank ID: virus name-country-year of collection or submission) are shown on the left. The pink and grey blocks represent the genomic regions from representative minor and major parent viruses, respectively. The numbers on the top of filled blocks indicate the nucleotide positions of breakpoints relative to the corresponding recombinant viruses on the left.

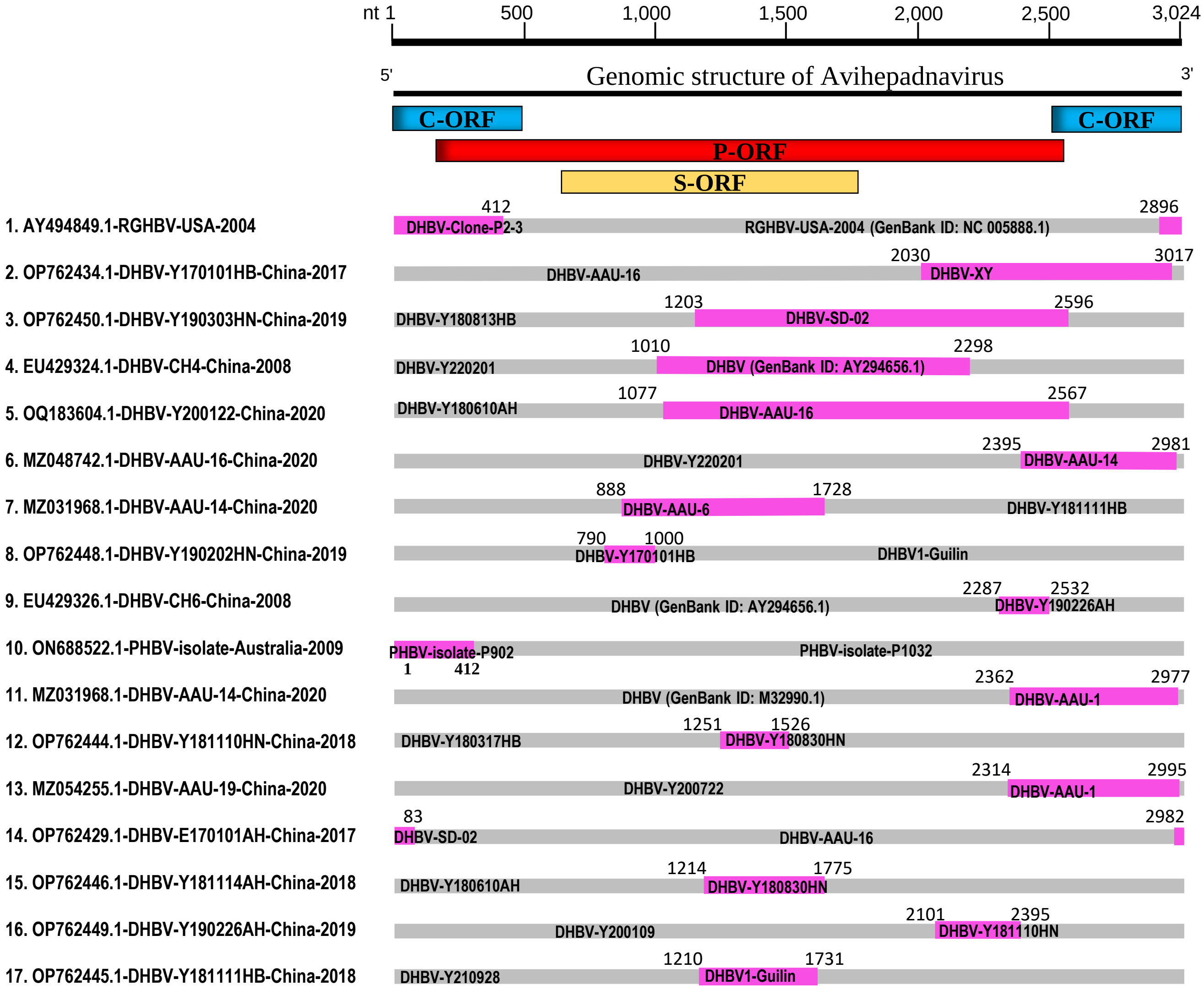

Supplementary Figure S4. Amino acid variability landscape of avihepadnavirus-encoded P, C, and S proteins. The plots represent amino acid variations in P protein **(A)**, C protein **(B)**, and S protein **(C)**. The Wu-Kabat variability coefficient for each amino acid was determined using PVS server. Y-axes represents the Wu-Kabat variability coefficient values, where the estimation limit is 1. Above the limit of 1 represents variation. X-axes represents the amino acid positions. The variability coefficient was calculated using following equation: variability = n*k/N. The n represents the number of sequences in the alignment, k is the number of different amino acids at a given position, and N is the time available for the most frequently identified amino acids.

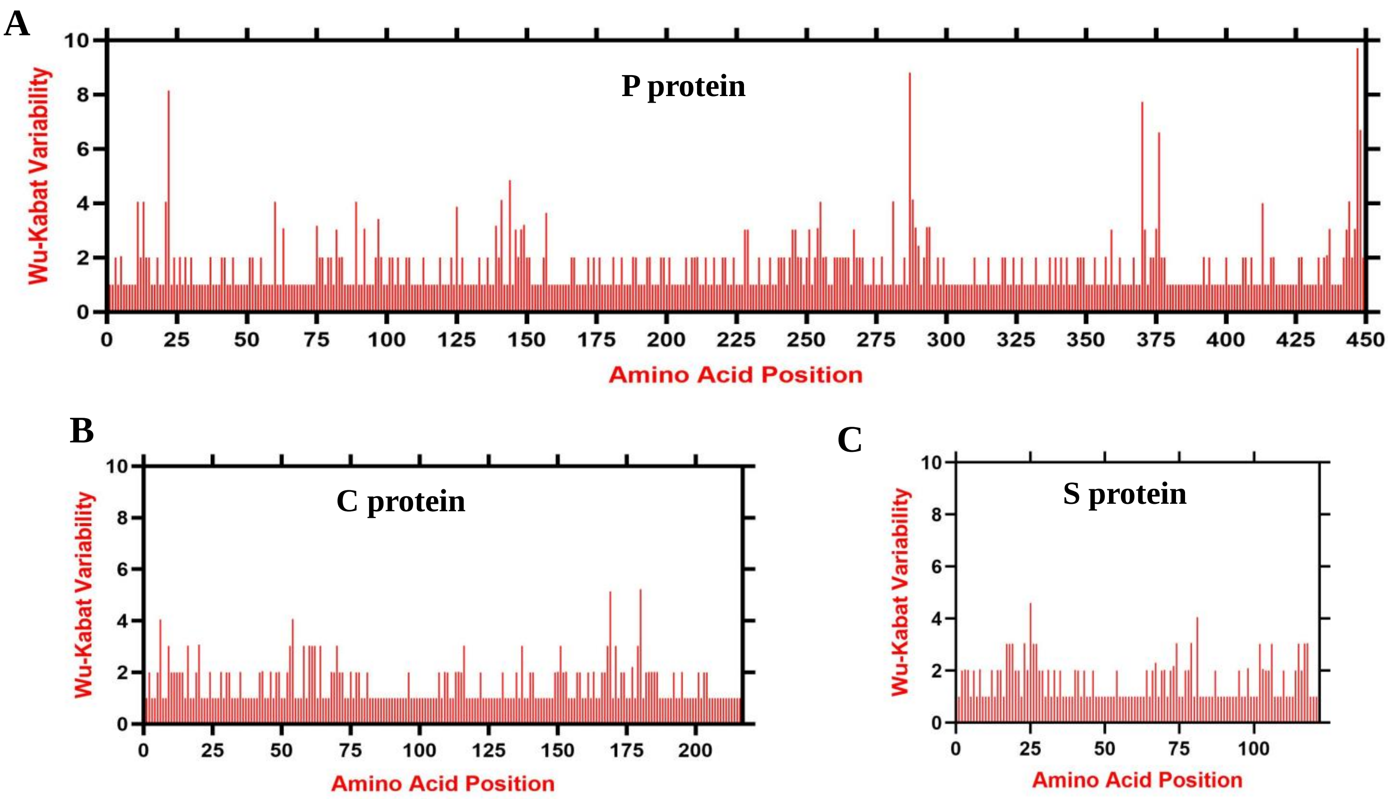

Supplement: Supplementary file 1 [file Data_Sheet_1.PDF]
