# Supplementary material for: Genetic diversity and phylogeographic dynamics of avihepadnavirus: a comprehensive full-length genomic view
Source: Front Vet Sci. 2024 May 2;11:1385033. doi: 10.3389/fvets.2024.1385033 (PMC11096447; doi:10.3389/fvets.2024.1385033)
Supplement: Supplementary file 2 [file Data_Sheet_2.PDF]

Supplementary Tables S1 and S2

Genetic Diversity and Phylogeographic Dynamics of Avihepadnavirus: A Comprehensive Full-length Genomic View

Running title: Avihepadnavirus evolution

Muhammad Sikandar[1#], Pir Tariq Shah[2,3#], Li Xing[1,4]*

Supplementary Table S1. Geographic distribution of avihepadnavirus genotype and sub-genotypes.

Genotype/ Sub-genotype			China	Germany	USA	France	South Africa	Australia	India	Canada	Poland	Total
GI	DHBV-I	a	36	1	3	3	6	-	1	1	-	51
		b	40	1	-	-	-	1	-	-	-	42
	SGHBV (DHBV-II)		-	5	-	-	-	-	-	-	-	5
	DHBV-III		-	-	4	-	-	-	-	-	-	4
	RGHBV		-	-	3	-	-	-	-	-	-	3
	CHBV		-	3	-	-	-	-	-	-	-	3
	ETHBV		-	3	-	-	-	-	-	-	-	3
	STHBV		-	3	-	-	-	-	-	-	-	3
	HHBV		-	-	1	-	-	-	-	-	-	1
GII	PHBV		-	-	-	-	-	1	-	-	20	21
											Total	136

Supplementary Table S2. The potential recombination events in the genome of avihepadnavirus.

Event serial NO.	Recombinant		Representative Minor parent		Representative Major parent		Detection methods						
	GenBank ID: Virus name-Country-Year of collection or submission	Genotype/Sub-genotype	GenBank ID: Virus name-Country-Year of collection or submission	Genotype/Sub-genotype	GenBank ID: Virus name-Country-Year of collection or submission	Genotype/Sub-genotype	R	G	B	M	C	S	T
1	AY494849.1-RGHBV-Female-Mandarin-duck-USA-2003	GI / RGHBV	M60677.1-DHBV-Clone-P2-3-USA-1993	GI / DHBV-Ia	NC005888.1-RGHBV-Ross-Goose-Hepatitis-B-Virus-USA-2004	GI / RGHBV	+	+	+	+	+	+	+
2	OP762434.1-DHBV-Y170101HB-China-2017	GI / DHBV-Ia	HQ214130.1-DHBV-XY-Henan-China-2010	GI / DHBV-Ia	MZ048742.1-DHBV-AAU-16-China-2020	GI / DHBV-Ia	+	+	+	+	+	+	+
3	OP762450.1-DHBV-Y190303HN-China-2019	GI / DHBV-Ia	MF471769.1-DHBV-SD-02-China-2016	GI / DHBV-Ia	OP762441.1-DHBV-Y180813HB-China-2018	GI / DHBV-Ib	+	+	+	+	+	+	+
4	EU429324.1-DHBV-CH4-Anas-platyrhynchos-China-2008	GI / DHBV-Ia	AY294656.1-DHBV-China-2003	GI / DHBV-Ia	OQ183601.1-DHBV-Y220201-China-2022	GI / DHBV-Ia	+	+	+	+	−	+	+
5	OQ183604.1-DHBV-Y200122-China-2022	GI / DHBV-Ib	MZ048742.1-DHBV-AAU-16-China-2020	GI / DHBV-Ia	OP762436.1-DHBV-Y180610AH-China-2018	GI / DHBV-Ib	+	+	+	+	+	+	+
6	*MZ048742.1-DHBV-AAU-16-China-2020	GI / DHBV-Ia	MZ031968.1-DHBV-AAU-14-China-2020	GI / DHBV-Ib	OQ183601.1-DHBV-Y220201-China-2022	GI / DHBV-Ia	+	+	+	+	+	+	+
7	MZ031968.1-DHBV-AAU-14-China-2020	GI / DHBV-Ib	MZ048741.1-DHBV-AAU-6-China-2020	GI / DHBV-Ia	OP762445.1-DHBV-Y181111HB-China-2018	GI / DHBV-Ib	+	+	+	+	+	+	+
8	OP762448.1-DHBV-Y190202HN-China-2019	GI / DHBV-Ia	JX469896.1-DHBV1-Guilin-China-2011	GI / DHBV-Ib	OP762434.1-DHBV-Y170101HB-China-2017	GI / DHBV-Ia	+	+	+	+	+	+	+
9	EU429326.1-DHBV-CH6-Anas-platyrhynchos-China-2008	GI / DHBV-Ia	OP762449.1-DHBV-Y190226AH-China-2019	GI / DHBV-Ia	AY294656.1-DHBV-China-2003	GI / DHBV-Ia	−	+	+	+	+	+	+
10	ON688522.1-PHBV-isolate-Australia-2009	GII / PHBV	JX274018.1-PHBV-isolate-P902-Poland-2009	GII / PHBV	JX274020.1-PHBV-isolate-P1032-Poland-2011	GII / PHBV	+	+	+	+	+	−	+
11	MZ031968.1-DHBV-AAU-14-China-2020	GI / DHBV-Ib	MZ031969.1-DHBV-AAU-1-China-2020	GI / DHBV-Ia	M32990.1-DHBV-Brown-Shanghai-duck-China--1993	GI / DHBV-Ib	+	+	+	+	+	+	+
12	OP762444.1-DHBV-Y181110HN-China-2018	GI / DHBV-Ib	OP762442.1-DHBV-Y180830HN-China-2018	GI / DHBV-Ia	OP762435.1-DHBV-Y180317HB-China|2018	GI / DHBV-Ib	+	+	+	+	−	+	+
13	MZ054255.1-DHBV-AAU-19-China-2020	GI / DHBV-Ia	MZ031969.1-DHBV-AAU-1-China-2020	GI / DHBV-Ia	OQ183607.1-DHBV-Y200722-China-2020	GI / DHBV-Ia	+	+	+	+	+	+	+
14	OP762429.1-DHBV-E170101AH-Goose-China-2017	GI / DHBV-Ia	MF471769.1-DHBV-SD-02-China-2016	GI / DHBV-Ia	MZ048742.1-DHBV-AAU-16-China-2020	GI / DHBV-Ia	+	+	−	+	+	−	+
15	OP762446.1-DHBV-Y181114AH-China-2018	GI / DHBV-Ib	OP762442.1-DHBV-Y180830HN-China-2018	GI / DHBV-Ia	OP762436.1-DHBV-Y180610AH-China-2018	GI / DHBV-Ib	+	+	+	+	+	+	+
16	OP762449.1-DHBV-Y190226AH-China-2019	GI / DHBV-Ia	OP762444.1-DHBV-Y181110HN-China-2018	GI / DHBV-Ib	OQ183599.1-DHBV-Y200109-China-2020	GI / DHBV-Ia	+	+	+	+	−	+	+
17	*OP762445.1-DHBV-Y181111HB-China-2018	GI / DHBV-Ia	JX469896.1-DHBV1-Guilin-China-2011	GI / DHBV-Ib	OQ183600.1-DHBV-Y210928-China-2021	GI / DHBV-Ib	+	+	−	+	+	+	+

R, RDP; G, GENECONV; B, BootScan; M, MaxChi; C, Chimaera; S, SiScan; T, 3seq. +, verified; -, not verified.

* The representative major or minor parent may be the actual recombinant due to the possibility of misidentification.
